# Supplementary material for: Causal Relationship between Aging and Anorexia Nervosa: A White-Matter-Microstructure-Mediated Mendelian Randomization Analysis
Source: Biomedicines. 2024 Aug 16;12(8):1874. doi: 10.3390/biomedicines12081874 (PMC11351342; doi:10.3390/biomedicines12081874)
Supplement: Supplementary file 1 [file biomedicines-12-01874-s001.zip › Supplementary Figure/Figure S8/Leaveoneout plot of MR analysis for TL to ubm-b-1912.pdf]

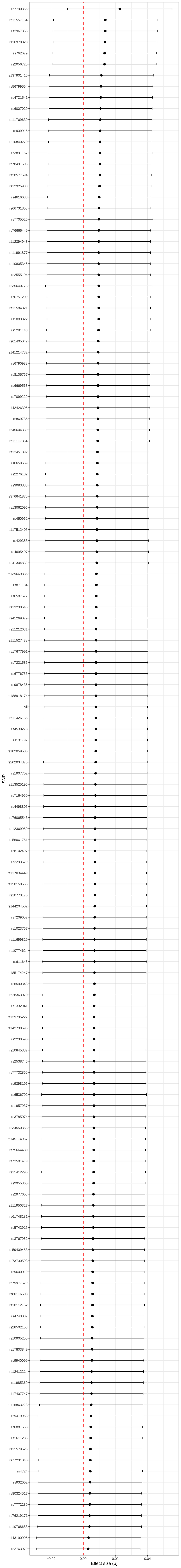
rs7790856
rs11557154
rs2967355
rs16978028
rs762679
rs2056726
rs137901416
rs56799554
rs4731541
rs6007020
rs11769630
rs939916
rs10840270
rs3891167
rs78491606
rs28577594
rs12925933
rs4616688
rs66731853
rs7705526
rs76666449
rs112394943
rs11991877
rs10805346
rs2555104
rs35640778
rs6751209
rs11584821
rs1003322
rs1291143
rs61405042
rs141214782
rs6790988
rs8105767
rs6669563
rs7099229
rs142426306
rs869785
rs45604339
rs11117354
rs12451892
rs6659669
rs2276182
rs3093888
rs376641875
rs13062095
rs450962
rs117512405
rs429358
rs4695407
rs41304832
rs139669835
rs871134
rs6587577
rs13230646
rs41269079
rs11212631
rs111527438
rs17677991
rs7221585
rs6776756
rs9878436
rs188918174
All
rs11426156
rs4530278
rs131797
rs182059586
rs202034370
rs1907702
rs113525195
rs7164950
rs4498805
rs76065543
rs12369950
rs56061761
rs8102497
rs2293579
rs117034449
rs150150565
rs10773176
rs144204502
rs7209057
rs1023767
rs11699829
rs10774624
rs611646
rs185174247
rs6590343
rs28363070
rs1332941
rs139795227
rs142730696
rs2230590
rs10845387
rs2538745
rs77732866
rs9398196
rs6536702
rs1957937
rs3785074
rs34550383
rs145114957
rs75664430
rs73581419
rs11412296
rs9955360
rs2977608
rs111950327
rs61748181
rs5742915
rs3767952
rs59409453
rs73730598
rs9600019
rs79977579
rs80116508
rs10112752
rs4743037
rs28502153
rs10905255
rs17803849
rs9940099
rs12412214
rs1985369
rs117407747
rs116863223
rs9419958
rs6881568
rs1611236
rs11579626
rs77231040
rs4724
rs932002
rs80324517
rs7772289
rs76219171
rs10768683
rs143190905
rs2763979
SNP
−0.02
0.00
0.02
0.04
Effect size (b)
